# Effects of Weight Loss in Metabolically Healthy Obese Subjects after Laparoscopic Adjustable Gastric Banding and Hypocaloric Diet

**DOI:** 10.1371/journal.pone.0017737

**Published:** 2011-03-08

**Authors:** Giorgio Sesti, Franco Folli, Lucia Perego, Marta Letizia Hribal, Antonio E. Pontiroli

**Affiliations:** 1 Department of Experimental and Clinical Medicine, University Magna Græcia of Catanzaro, Catanzaro, Italy; 2 Diabetes Division, Department of Medicine, University of Texas Health Science Center, San Antonio, Texas, United States of America; 3 Dipartimento di Medicina, Chirurgia e Odontoiatria, University of Milano, Milan, Italy; University of Padova, Medical School, Italy

## Abstract

Weight loss in metabolically healthy obese (MHO) subjects may result in deterioration of cardio-metabolic risk profile. We analyzed the effects of weight loss induced by laparoscopic adjustable gastric banding (LAGB) on cardio-metabolic risk factors in MHO and insulin resistant obese (IRO) individuals. This study included 190 morbidly obese non-diabetic subjects. Obese individuals were stratified on the basis of their insulin sensitivity index (ISI), estimated from an OGTT, into MHO (ISI index in the upper quartile) and IRO (ISI in the three lower quartiles). Anthropometric and cardio-metabolic variables were measured at baseline and 6-months after LAGB. Six months after LAGB, anthropometric measures were significantly reduced in both MHO and IRO. Percent changes in body weight, BMI, and waist circumference did not differ between the two groups. Fasting glucose and insulin levels, triglycerides, AST, and ALT were significantly reduced, and HDL cholesterol significantly increased, in both MHO and IRO subjects with no differences in percent changes from baseline. Insulin sensitivity increased in both MHO and IRO group. Insulin secretion was significantly reduced in the IRO group only. However, the disposition index significantly increased in both MHO and IRO individuals with no differences in percent changes from baseline between the two groups. The change in insulin sensitivity correlated with the change in BMI (r = −0.43; *P*<0.0001). In conclusion, our findings reinforce the recommendation that weight loss in response to LAGB intervention should be considered an appropriate treatment option for morbidly obese individuals regardless of their metabolic status, i.e. MHO vs. IRO subjects.

## Introduction

Increasing evidence suggests that a subset of relatively insulin sensitive obese subjects, referred to as metabolically healthy obese (MHO) individuals, has a favorable cardio-metabolic risk profile and a lower risk for type 2 diabetes and cardio-vascular disease, as compared with insulin resistant obese (IRO) subjects [1], [2]. It is worth noting that the MHO phenotype is observed in a given moment of the natural history of obesity, but it is unknown whether MHO individuals will maintain their metabolic status or develop over time the IRO phenotype. Whether the MHO phenotype is associated with a lower risk for all-cause mortality is an object of debate with divergent results reported by two recent studies [3], [4]. Mechanistically, several features might contribute to the favorable cardio-metabolic profile of MHO individuals including lower visceral adipose tissue accumulation, lower ectopic fat accumulation in skeletal muscle and liver, lower blood pressure, lower levels of triglycerides, free fatty acids, liver enzymes (as a surrogate markers of fatty liver), inflammatory markers, higher beta-cell function, higher estimated glomerular filtration rate, and higher levels of HDL cholesterol, insulin-like growth factor 1 (IGF-1), and adiponectin [5]–[11].

A controversial question is whether MHO individuals would have any cardio-metabolic benefit from weight loss. Studies assessing the effects of lifestyle interventions, including diet and/or exercise, in MHO individuals have led to divergent results [12]–[15]. Three studies have reported an improvement in the cardio-metabolic risk profile in IRO, but not in MHO, subjects, despite similar weight loss [12]–[14], whereas one study has shown an improvement in cardio-metabolic risk factors of MHO individuals after a weight loss intervention [15]. Laparoscopic adjustable gastric banding (LAGB) has been proposed as a therapeutic approach to achieve durable weight loss in morbidly obese subjects [16]. Because bariatric surgery-induced weight loss is associated with an improvement of the cardio-metabolic risk profile in morbidly obese subjects [16], [17], we sought to investigate the effects of LAGB-induced weight loss on cardio-metabolic risk factors among MHO and IRO individuals.

## Materials and Methods

### Subjects

The study group consisted of 190 morbidly obese non-diabetic subjects consecutively recruited at the Istituto Clinico Sant'Ambrogio, Ospedale San Paolo and Ospedale San Raffaele, Milano, Italy. All subjects were Caucasian. Eligibility criteria, time-schedules for assessments, and diet after LAGB have been described previously in detail [16]–[18]. All anthropometric and biochemical measurements were made in the morning after a 12-h fast using standardized methods. Height and weight were measured to the nearest 0.5 cm and 0.1 kg, respectively. Waist (at the midpoint between the lateral iliac crest and lowest rib) was measured to the nearest 0.5 cm. A 75 g oral glucose tolerance test (OGTT) was performed with sampling for plasma glucose and insulin. After LAGB, subjects were reevaluated by a dietitian and a physician at fortnight intervals for 2 months and then monthly up to 6 months, as previously described [16]–[18]. The study was approved by the Istituto San Raffaele Ethics Committee and by the Review Board of the University "Magna Graecia" of Catanzaro, and informed written consent was obtained from each participant.

### Analytical determinations

Plasma glucose was measured by the glucose oxidation method (YSI, Inc., Yellow Springs, OH). Triglyceride levels were assayed by an enzymatic technique on a Cobas Fara II Centrifugal Analyser (Cobas Fara II; Roche, Basel, Switzerland). Total cholesterol and HDL-cholesterol levels were assayed by enzymatic automated spectrophotometric methods with a Cobas Fara II. Insulin was assayed by a Microparticle Enzyme Immunoassay (IMX; Abbott Laboratories, Abbott Park, IL) with a monoclonal antibody without cross-reactivity with human proinsulin. Alanine aminotransferase (ALT) and aspartate aminotransferase (AST) were determined by standard laboratory methods.

### Calculations

A previously validated index (ISI) derived from the OGTT [19] was used to estimate insulin sensitivity. Men and women separately were stratified into quartiles according to their insulin sensitivity estimated from the OGTT results. Men and women in the upper quartile were then combined and were defined as MHO, while men and women in the three other quartiles were defined as being insulin resistant obese (IRO). Glucose-stimulated insulin secretion was estimated by the Stumvoll index for first phase insulin secretion as previously described [20]. To evaluate β-cell function, the so called disposition index was calculated as the product of the ISI and the Stumvoll index for first phase insulin secretion. Change in variables at the 6-month follow-up were calculated as follows: ([Variable_6-month follow-up_ − Variable_baseline_] / Variable_baseline_) × 100.

### Statistical analysis

Triglycerides, ALT, AST, fasting insulin and insulin values during OGTT were natural log transformed for statistical analysis due to their skewed distribution. Continuous data are expressed as means ± SD. Categorical variables were compared by χ2 test. In the within-group test, differences between baseline and follow-up variables were tested by paired t-test. In the between-groups test, differences in variables between the two groups (MHO vs. IRO group) were analyzed using Student's t-test. Relationships between changes of variables were determined by Pearson's correlation coefficient (r). A *P* value <0.05 was considered statistically significant. All analyses were performed using the SPSS software programme Version 16.0 for Windows.

## Results

### Baseline study

The clinical characteristics and laboratory data for MHO and IRO subjects at baseline are shown in Table 1. By design, insulin sensitivity, measured by ISI, was higher in MHO subjects (108±31) as compared with IRO individuals (47±18; *P*<0.0001). The ISI value of MHO subjects was similar to the mean value obtained for 332 non-obese individuals (128 men and 204 women; age = 42±14, BMI = 23.8±2.2, ISI = 114±54) participating to the to the CAtanzaro MEtabolic RIsk factors (CATAMERI) Study, a cross-sectional study assessing cardio-metabolic risk factors in an ambulatory care setting [21], [22]. No statistically significant differences in gender, age, total cholesterol, triglycerides, HDL cholesterol, and AST were observed between the two groups of obese individuals. IRO subjects showed significantly higher body weight, BMI, waist circumference, fasting and 2-h glucose levels, fasting and 2-h insulin levels, ALT, and insulin secretion index as compared with MHO subjects. Since insulin secretion is dependent on the actual insulin sensitivity, we compared the disposition index, an integrated measure of the ability of the β-cells to compensate for insulin resistance, between the two groups. IRO individuals exhibited a lower disposition index as compared with MHO subjects, suggesting that the IRO phenotype is associated with a decreased ability to compensate for insulin resistance.

**Table 1:** Clinical characteristics and laboratory data at baseline and after LAGB intervention in MHO and IRO subjects.

Variables	MHO Baseline	MHO 6-months follow-up	^§^ *P*	IRO Baseline	IRO 6-months follow-up	^§^ *P*
Male/Female	8/40	---		40/119	---	
Age (*years*)	38±10	---		40±10	---	
Body weight (*kg*)	108±16	92±14	<0.0001	118±20**	102±17***	<0.0001
BMI (*kg/m^2^*)	41.1±5.5	35.0±5.3	<0.0001	44.0±6.4**	38.2±5.6**	<0.0001
Waist circumference (*cm*)	115±12	100±10	<0.0001	123±13**	109±12***	<0.0001
Fasting Glucose (*mgl/dl*)	93±12	88±12	0.009	102±14***	95±12**	<0.0001
2 h glucose (*mgl/dl*)	105±20	101±30	0.32	130±39***	118±37**	0.001
Fasting Insulin *(µU/ml)*	11±3	8±3	0.001	20±12***	12±6***	<0.0001
2-h insulin (*µU/ml*)	27±17	31±23	0.32	78±57***	57±46***	<0.0001
Total cholesterol (*mg/dl*)	202±41	203±45	0.88	203±38	202±35	0.59
HDL (*mg/dl*)	48±12	52±12	0.02	49±12	51±13	0.002
Triglycerides (*mg/dl*)	119±53	100±46	<0.0001	140±69	115±49	<0.0001
ALT (*UI/l*)	28±14	22±14	0.009	36±25*	24±14	<0.0001
AST (*UI/l*)	22±7	19±7	0.005	26±15	19±8	<0.0001
ISI	108±31	141±51	0.02	47±18***	84±46***	<0.0001
Stumvoll first-phase index	1140±332	1142±395	0.96	1740±845***	1412±684*	<0.0001
Disposition index	121708±40793	153107±60566	0.01	73870±30773***	105675±54677**	<0.0001

Data are means ± SD. Triglycerides_,_ ALT, AST, fasting, and 2-h insulin were log transformed for statistical analysis, but values in the table represent a back transformation to the original scale. Categorical variables were compared by χ2 test. §*P* values for differences between baseline and follow-up variables tested by paired t-test. *P values for differences of continuous variables between the two groups using unpaired Student's t.

*P <0.01 vs. MHO,

**P <0.001 vs. MHO;

***P <0.0001 vs. MHO.

### Follow-up study after LAGB

**Figure 1:**
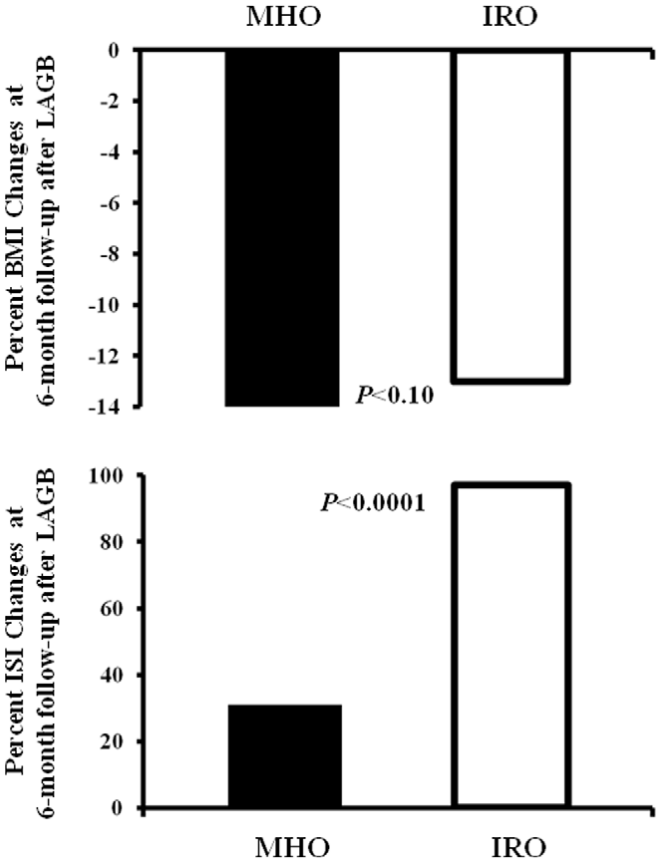
Change (%) in BMI and insulin sensitivity assessed as ISI at 6-month follow-up after laparoscopic adjustable gastric banding (LAGB) intervention in MHO and IRO subjects.

**Figure 2:**
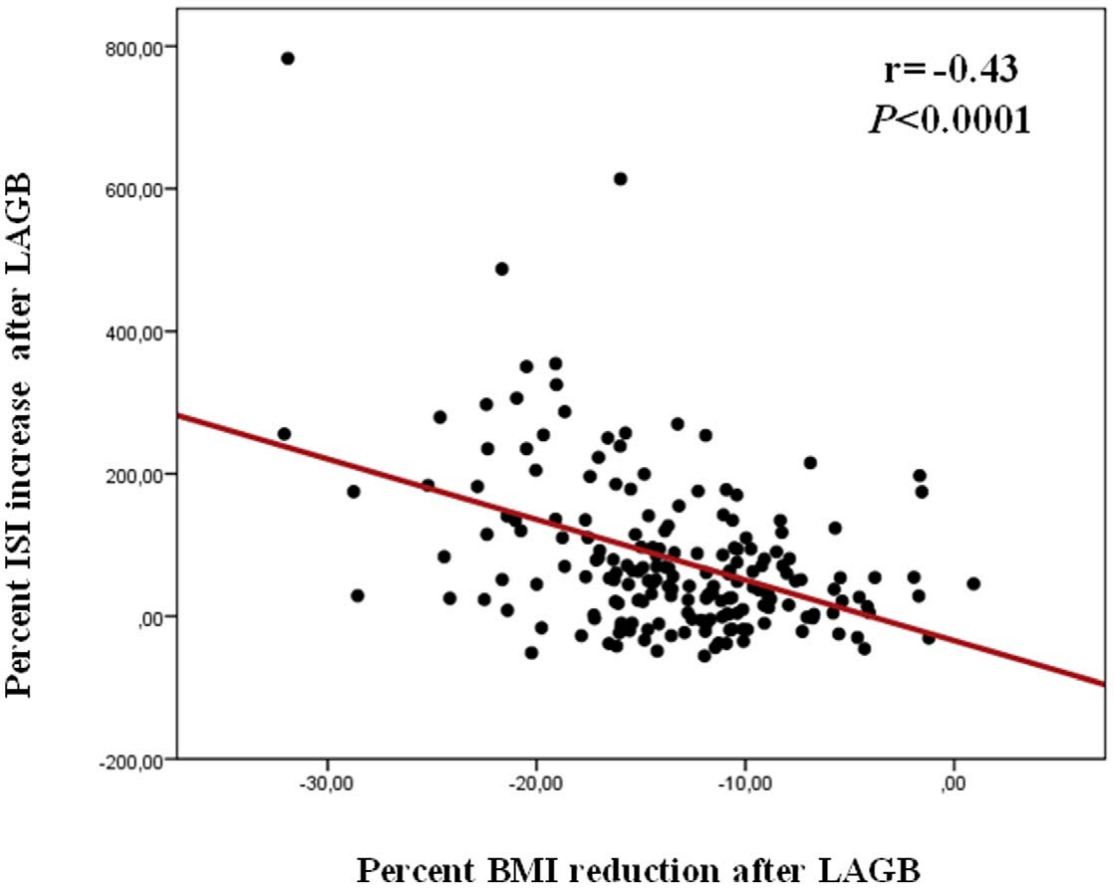
Correlation between percent changes in BMI and ISI after laparoscopic adjustable gastric banding (LAGB) intervention.

Six months after LAGB intervention, body weight, BMI, and waist circumference were significantly reduced in both MHO and IRO subjects. Percent changes in body weight, BMI (Figure 1), and waist circumference did not differ between MHO and IRO group (Table 2). Fasting glucose and insulin levels, triglycerides, AST, and ALT significantly reduced and HDL cholesterol significantly increased in both MHO and IRO subjects with no difference in percent changes from baseline between the two groups (Table 2). 2-h glucose and insulin levels were significantly reduced in the IRO group only. Insulin sensitivity, assessed by ISI, increased in both MHO and IRO group (Table 2); the percent change was significantly greater in the IRO group (Figure 1). Insulin secretion was significantly reduced in the IRO group, while no change was observed in the MHO group. However, the disposition index significantly increased in both MHO and IRO individuals with no significant difference in percent changes from baseline between the two groups. In both groups, the change in insulin sensitivity correlated with the change in BMI ( r =  −0.43; *P*<0.0001) (Figure 2). This correlation was stronger in the IRO group (r =  −0.49; *P*<0.0001) than in the MHO group (r =  −0.39; *P* = 0.005).

**Table 2:** Change (%) in clinical characteristics and laboratory data at 6-month follow-up after LAGB intervention in MHO and IRO subjects.

Variables	MHO Change (%) at 6-month follow-up	IRO Change (%) at 6-month follow-up	*P* for % change between the two
Body weight (*kg*)	−14±4	−13±6	0.10
BMI (*kg/m^2^*)	−14±4	−13±6	0.10
Waist circumference (*cm*)	−12±6	−10±5	0.08
Fasting Glucose (*mgl/dl*)	−6.7±2.5	−8.9±1.6	0.60
2 h glucose (*mgl/dl*)	−1.4±4.9	−4.0±2.8	0.65
Fasting Insulin (*µU/ml*)	−38±9	−91±11	0.006
2-h insulin (*µU/ml*)	−32±17	−105±15	0.008
Total cholesterol (*mg/dl*)	−1.0±1.8	−1.3±1.1	0.91
HDL (*mg/dl*)	5.4±3.0	3.7±1.7	0.62
Triglycerides (*mg/dl*)	−24±5	−25±6	0.85
ALT (*UI/l*)	−42±9	−59±8	0.11
AST (*UI/l*)	−16±4	−30±5	0.03
ISI	31±29	97±80	<0.0001
Stumvoll first-phase index	5±5	−8±5	0.11
Disposition index	8±5	18±15	0.20

Data are means ± SD. Triglycerides, ALT, AST, fasting, and 2-h insulin were log transformed for statistical analysis, but values in the table represent a back transformation to the original scale. Categorical variables were compared by χ2 test. §*P* values for differences between baseline and follow-up variables tested by paired t-test. *P values for differences of continuous variables between the two groups using unpaired Student's t. *P <0.01 vs. MHO, **P <0.001 vs. MHO; ***P <0.0001 vs. MHO.

## Discussion

Obesity has constantly been associated with cardio-metabolic abnormalities, including dysglycemia, insulin resistance, hypertension, dyslipidemia, which may increase the risk of cardiovascular diseases and type 2 diabetes. However, increasing evidence suggests that obesity is a heterogeneous disorder with a subset of obese subjects, known as metabolically healthy obese (MHO), that appears to be protected against obesity-related cardio-metabolic abnormalities [1]–[11]. Lifestyle intervention is considered the primary treatment strategy for all obese individuals; however, bariatric surgery has been successfully employed to achieve stable reduction of body weight in morbidly obese individuals [16], [17]. Recent studies have raised the question of whether MHO subjects may gain any additional cardio-metabolic benefit from weight loss; three studies have reported no change in cardio-metabolic risk profile or a paradoxical deterioration in insulin sensitivity in MHO subjects despite significant weight loss [12]–[14], whereas one study has shown an improvement in cardio-metabolic risk factors among MHO individuals after lifestyle intervention [15]. Our results are in agreement with the latter study as we observed that, six months after LAGB intervention, MHO individuals had a significant improvement in cardio-metabolic risk factors including an increase of insulin sensitivity, β-cell function, and HDL cholesterol, and a reductions of fasting glucose and insulin levels, triglycerides, and liver enzymes (surrogate markers of fatty liver). Importantly, to the best of our knowledge, this is the first study assessing the effects of LAGB intervention on MHO individuals. Several explanations may account for the differing results of the present study as compared with previous studies. The most obvious explanation is related to the greater reduction of body weight obtained after LAGB intervention as compared with lifestyle intervention. After a 6-months lifestyle intervention, weight loss in MHO individuals ranged from −5 kg to −2.4 kg [12]–[15], whereas, after LAGB intervention, body weight decreased by 16 kg in MHO individuals. Given that the reduction in BMI was strongly correlated with the increase in insulin sensitivity (Figure 1), it seems plausible that a critical amount of total adiposity must be lost before observing a beneficial effect of weight loss on insulin sensitivity. Other potential mechanisms explaining the present results include changes in ghrelin or incretins release.

Strengths of the present study include the relatively large sample size, the longitudinal design, the inclusion of both sexes, the well characterized cohort of morbidly obese individuals with simultaneous assessment of plasma glucose and insulin levels during an OGTT and cardio-metabolic risk factors, and the centralization of laboratory analyses. Nonetheless, the present study has some limitations. Most notably, all biochemical variables, including plasma glucose during OGTT were measured once, a common limitation to most epidemiological studies. In addition, the present findings are only based on Caucasian individuals, and results might vary as a function of ethnic group. Finally, we have not assessed cardiovascular function indexes, such as endothelial-mediated vasodilatation or arterial stiffness, that may be modified by weight loss.

As restricted health care resources lead to the need to prioritize high-risk obese individuals for aggressive treatment, including bariatric surgery, the notion that subjects with uncomplicated obesity would not benefit from weight loss seems a misguided message to both regulatory authorities and public opinion. Our findings reinforce the recommendation that weight loss in response to bariatric surgery intervention should be considered an appropriate treatment option for all morbidly obese individuals, regardless of their metabolic status, i.e. MHO vs. IRO subjects.
